# Overcoming BRAF and CDK4/6 inhibitor resistance by inhibiting MAP3K3-dependent protection against YAP lysosomal degradation

**DOI:** 10.1038/s12276-024-01210-5

**Published:** 2024-04-16

**Authors:** Sanghyun Park, Won-Ji Ryu, Tae Yeong Kim, Yumi Hwang, Hyun Ju Han, Jeong Dong Lee, Gun Min Kim, Joohyuk Sohn, Sang Kyum Kim, Min Hwan Kim, Joon Kim

**Affiliations:** 1 Department of Dermatology, Chonnam National University Medical School, Gwangju, Korea; 2 Graduate School of Medical Science and Engineering, Korea Advanced Institute of Science and Technology (KAIST), Daejeon, Korea; 3 Avison Biomedical Research Center, Yonsei University College of Medicine, Seoul, Korea; 4 Division of Medical Oncology, Department of Internal Medicine, Yonsei University College of Medicine, Seoul, Korea; 5 Department of Pathology, Yonsei University College of Medicine, Seoul, Korea

**Keywords:** Cancer therapeutic resistance, Cell growth

## Abstract

Transcriptional programs governed by YAP play key roles in conferring resistance to various molecular-targeted anticancer agents. Strategies aimed at inhibiting YAP activity have garnered substantial interest as a means to overcome drug resistance. However, despite extensive research into the canonical Hippo–YAP pathway, few clinical agents are currently available to counteract YAP-associated drug resistance. Here, we present a novel mechanism of YAP stability regulation by MAP3K3 that is independent of Hippo kinases. Furthermore, we identified MAP3K3 as a target for overcoming anticancer drug resistance. Depletion of MAP3K3 led to a substantial reduction in the YAP protein level in melanoma and breast cancer cells. Mass spectrometry analysis revealed that MAP3K3 phosphorylates YAP at serine 405. This MAP3K3-mediated phosphorylation event hindered the binding of the E3 ubiquitin ligase FBXW7 to YAP, thereby preventing its p62-mediated lysosomal degradation. Robust YAP activation was observed in CDK4/6 inhibitor-resistant luminal breast cancer cells. Knockdown or pharmacological inhibition of MAP3K3 effectively suppressed YAP activity and restored CDK4/6 inhibitor sensitivity. Similarly, elevated MAP3K3 expression supported the prosurvival activity of YAP in BRAF inhibitor-resistant melanoma cells. Inhibition of MAP3K3 decreased YAP-dependent cell proliferation and successfully restored BRAF inhibitor sensitivity. In conclusion, our study reveals a previously unrecognized mechanism for the regulation of YAP stability, suggesting MAP3K3 inhibition as a promising strategy for overcoming resistance to CDK4/6 and BRAF inhibitors in cancer treatment.

## Unveiling MAP3K3 as a Key Regulator of YAP Activity

Cancer treatment often uses specific drugs, but sometimes the body can resist these, causing the cancer to return. A study led by Yonsei University College of Medicine researchers looked at the role of a protein, yes-associated protein (YAP), in this drug resistance. They found that another protein, MAP3K3, helps keep YAP stable, which then helps the body resist cancer drugs. By stopping MAP3K3, the researchers could lower YAP levels and overcome drug resistance in breast cancer and skin cancer cells. This suggests that focusing on MAP3K3 could be a good way to treat cancers that resist drugs. The study also shows how important it is to understand how proteins in cancer cells interact.

This summary was initially drafted using artificial intelligence, then revised and fact-checked by the author.

## Introduction

Therapeutic agents targeting oncogenic drivers, such as BRAF inhibitors in melanoma and CDK4/6 inhibitors in breast cancer, are currently established as the mainstays of cancer therapy. However, adaptive or acquired resistance eventually causes relapse from long-lasting tumor regression, leading to the repopulation of resistant cancer clones. Recently, yes-associated protein (YAP) has been established as a versatile mediator of resistance to molecular targeted therapeutic agents, such as epidermal growth factor receptor (EGFR), BRAF, and cyclin-dependent kinase (CDK)-4/6 inhibitors, as well as anti-human epidermal growth factor receptor 2 (HER2) antibodies, by contributing to cell cycle progression and antiapoptotic pathway activation^1–3^. Thus, the YAP pathway can be therapeutically targeted to overcome resistance to a wide range of anticancer drugs. Pharmacological strategies to suppress YAP activity in cancers, such as inhibition of YAP-regulating kinases^4,5^ or direct inhibition of YAP-TEAD binding, have been proposed^6,7^. However, few anticancer drugs that modulate YAP transcriptional activity have been clinically validated.

The cellular activity of YAP is determined by its nuclear localization and protein stability^8^. Large tumor suppressor (LATS) kinases, the main effectors of the Hippo signaling cascade, phosphorylate YAP and induce its cytoplasmic retention as well as its polyubiquitination and proteasomal degradation^9,10^. In addition to the Hippo pathway, as the primary pathway for YAP regulation, several Hippo-independent regulators also act in diverse cellular contexts and include osmotic stress-mediated NLK activation and the regulation of YAP permeability through nuclear pores^11–14^. Members of the mitogen-activated protein kinase (MAPK) superfamily are involved in multiple signaling pathways governing a diverse array of cellular processes, including cell proliferation, differentiation, and death. Importantly, several MAP kinases have been reported to regulate the activity of the Hippo–YAP pathway. MAP4Ks phosphorylate LATS1/2 in parallel with MST1/2, thus inhibiting YAP activity^15,16^. MAP2K1 controls the YAP protein level by phosphorylating beta-transducin repeat-containing E3 ubiquitin protein ligase (β-TrCP), which can protect YAP from degradation^17^. JNK and p38 also promote nuclear YAP translocation downstream of the actin cytoskeleton in mouse alveolar stem cells^18^, and ERK induces YAP transcription by stabilizing cAMP response element-binding protein (CREB), whereas p38 inhibits CREB-mediated YAP transcription^19^.

MAP3K3 (also called MEKK3) is a serine/threonine kinase of the MAPK superfamily that acts upstream of the ERK5 and p38 pathways^20,21^. MAP3K3 overexpression promotes apoptosis resistance by elevating NF-kB activity and the expression of survival factors^22^. Moreover, MAP3K3 upregulation is associated with poor survival in esophageal squamous cell carcinoma, cervical cancer, and ovarian cancer^23–25^. A recent study reported the requirement of MAP3K3 for YAP target gene transcription in pancreatic cancer cells^26^. However, the molecular mechanism underlying the regulation of YAP by MAP3K3 has not been determined.

Our previous RNAi screening studies suggested that MAP3K3 is a potential target for YAP inhibition^5,27^. In this study, we demonstrate that MAP3K3 phosphorylates YAP at serine 405 and that this phosphorylation event interferes with YAP polyubiquitination and lysosomal degradation in a LATS-independent manner. Moreover, we show that MAP3K3 inhibition, achieved through RNAi-mediated knockdown or pharmacological means, overcomes CDK4/6 inhibitor resistance in luminal breast cancer cells. We also report a correlation between MAP3K3 expression and the YAP protein abundance in cutaneous melanoma, and we demonstrate the efficient suppression of YAP-associated BRAF inhibitor resistance through MAP3K3 inhibition. Collectively, our results suggest that MAP3K3 is a promising target for the development of therapeutic agents to overcome BRAF and CDK4/6 inhibitor resistance as well as to treat other YAP-mediated malignant pathologies.

## Materials and methods

### Cell culture

Human RPE1, HEK293T, MCF7, T47D, and SKMEL28 cells were acquired from the American Type Culture Collection (ATCC). WM3248 cells were acquired from the Coriell Institute. LATS1/2 DKO HEK293A cells were established by the laboratory of Kun-Liang Guan at UC San Diego. Large frozen cell stocks were generated to prevent contamination with other cell lines. All cell lines were used within 10 passages after the revival of the frozen stock cultures. The cells were free of mycoplasma contamination, as determined by DAPI staining every two to three passages. RPE1 cells were maintained in Dulbecco’s modified Eagle’s medium (DMEM)/F-12 medium (Welgene) supplemented with 10% fetal bovine serum (FBS; Welgene). HEK293T cells were maintained in DMEM (Welgene) supplemented with 10% FBS. SKMEL28 cells were maintained in Eagle’s minimum essential medium (Welgene) supplemented with 10% FBS. WM3248 cells were maintained in a 4:1 mixture of MCDB-153 medium and L-15 medium (Welgene) supplemented with 2%

FBS, 1.68 mM CaCl_2_, and 5 μg/ml insulin (Welgene). MCF7 and T47D cells were maintained in RPMI 1640 medium (Gibco) supplemented with 10% FBS. All media contained 1% penicillin‒streptomycin (Sigma Aldrich). Vemurafenib (PLX4032)-resistant SKMEL28 (SKMEL28_VemR) cells were generated in our previous study^28^ and maintained in the presence of vemurafenib (2 μM). Palbociclib-resistant cells (MCF7_PalR and T47D_PalR cells) were generated from parental MCF7 and T47D cells through treatment with palbociclib at stepwise concentrations (100 nM to 400 nM) for six months. MCF7_PalR and T47D_PalR cells were maintained in the presence of palbociclib (400 nM).

### Plasmids and transfection

The plasmid vectors used in this study are described in Supplementary Table 1. The expression plasmids for YAP mutants (YAP S405A, YAP S405D, and YAP S403D) and kinase-dead MAP3K3 (MAP3K3 K391A)^29^ were generated using a QuikChange Lightning Site-Directed Mutagenesis Kit (Agilent Technologies) according to the manufacturer’s protocol. Cell transfection with plasmid vectors and siRNAs was performed using Lipofectamine LTX with PLUS Reagent (Invitrogen) and Lipofectamine RNAiMAX Reagent (Invitrogen), respectively. For lentiviral particle assembly, the pLKO.1-TRC lentiviral construct, a packaging plasmid (psPAX2), and an envelope plasmid (pMD2.G) were cotransfected into HEK293T cells, and lentiviral supernatants were collected 36 h after transfection. Each supernatant was filtered through a 0.45-μm filter and used to infect SKMEL28_VemR cells and MCF7_PalR cells in the presence of 8 μg/ml polybrene (Sigma Aldrich). Infected cells were selected using puromycin (2 μg/ml).

### Immunoblotting and immunoprecipitation

For immunoblotting, cells were lysed using RIPA lysis buffer supplemented with protease and phosphatase inhibitor cocktails (Sigma Aldrich). The cell lysates were centrifuged for 5 min at 4 °C and 13,000 × *g*. Proteins in the supernatants (10–40 μg) were separated via sodium dodecyl sulfate–polyacrylamide gel electrophoresis (SDS–PAGE) and transferred to nitrocellulose or polyvinylidene fluoride (PVDF) membranes. Phosphorylation was analyzed using Phos-tag acrylamide (Wako) according to the manufacturer’s protocol. Lysate fractions were prepared using NE-PER Nuclear and Cytoplasmic Extraction Reagents (Thermo Fisher Scientific) according to the manufacturer’s protocol. For immunoprecipitation, NP-40 lysis buffer was used for cell lysis. Cell lysates (200–500 µg) were incubated with the antibodies indicated in the figures and with protein G agarose beads. The beads were washed three times with lysis buffer and centrifuged at 3000 × *g* for 30 s. Equal amounts of protein were denatured by boiling in Laemmli sample buffer, separated via SDS‒PAGE, and transferred to nitrocellulose or PVDF membranes. Polyclonal anti-phospho-YAP serine 405 antibodies were generated using the YAP peptide GLSM(pS)SYSVPR and affinity purified (Youngin Frontier). The other antibodies used in this study are described in Supplementary Table 1.

### In vitro kinase assay

Recombinant human YAP1 (Abcam) and MAP3K3 (Abcam) proteins were incubated in kinase buffer containing 100 mM Tris-HCl (pH 7.4), 10 mM MgCl_2_, and 100 μM ATP at 30 °C for 30 min. The reaction was stopped by the addition of Laemmli buffer. Proteins were separated via SDS‒PAGE for analysis by immunoblotting.

### Public gene expression data analysis

Gene expression data were obtained from tumor biopsies included in the NeoPalAna trial (GSE93204)^30^ in the Gene Expression Omnibus (GEO) database. MAP3K3 expression and the YAP-dependent expression signature (YAP1_UP in MSigDB C6) were compared between the baseline and on-treatment samples. The YAP-dependent expression signature was calculated using the gene set variation analysis algorithm^31^.

### RNA sequencing and data analysis

Total RNA was extracted from cells using an RNeasy Plus Mini Kit (QIAGEN) according to the manufacturer’s protocol. The RNA sequencing library was prepared using a TruSeq Stranded mRNA LT Sample Prep Kit (Illumina), and sequencing was performed using a NovaSeq 6000 system (Illumina) to generate 101-bp paired-end reads. FastQC v0.11.7 and Trimmomatic 0.38 were used to filter out low-quality sequencing reads, and the retained reads were mapped to the human genome UCSC hg19 using Bowtie2 2.3.4.1 and HISAT2 version 2.1.0. StringTie version 1.3.4d was used to generate the raw read counts. Gene set enrichment analysis (GSEA)^32^ was performed using the C6 MsigDB gene set database to identify enriched oncogenic signature gene sets.

### Statistical analysis

All the data analyses conducted in this study were performed using GraphPad Prism 7 for Windows. Quantitative analysis was performed for at least three independent experimental groups. Statistical analysis of differences between groups was performed using two-tailed Student’s *t* test to determine significance. The chi-square test was used to determine whether there was a significant association between two categorical variables. *p* < 0.05 was considered to indicate statistical significance. The error bars in all the graphs indicate the standard error of the mean (SEM) values.

## Results

### MAP3K3 maintains YAP protein stability independent of LATS1/2-mediated proteasomal degradation

**Fig. 1:**
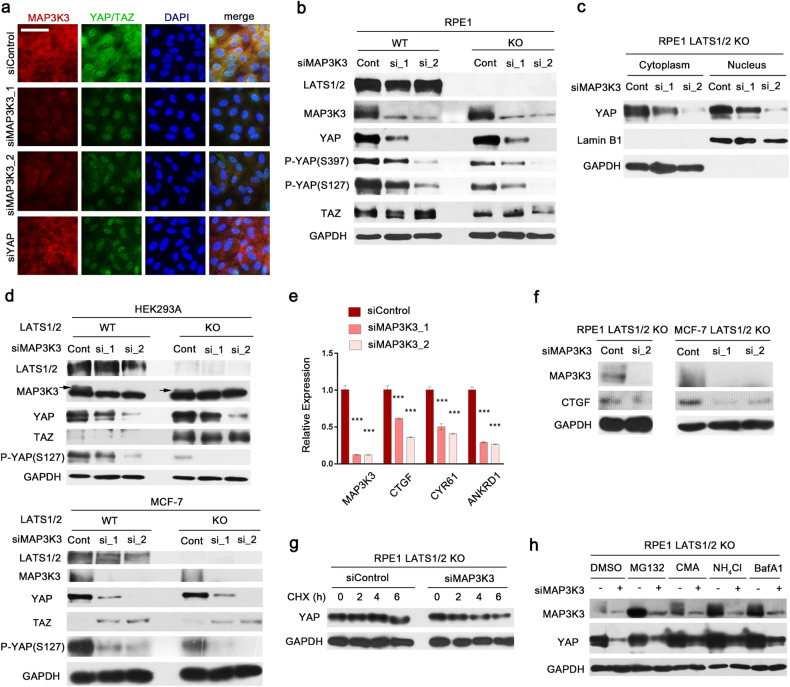
MAP3K3 contributes to YAP protein stability. **a** Immunofluorescence (IF) micrographs showing decreases in the levels of nuclear and cytoplasmic YAP in LATS1/2-null RPE1 cells after MAP3K3 knockdown using two distinct siRNAs. YAP knockdown was conducted as a control for evaluating YAP downregulation. Scale bar, 50 μm. **b** Immunoblot (IB) analysis of the indicated proteins in LATS1/2-wild-type and LATS1/2-null RPE1 cells after MAP3K3 knockdown. **c** IB analysis of YAP in LATS1/2-null RPE1 cells. Cell lysates were separated into the nuclear and cytoplasmic fractions. Lamin B1 and GAPDH were used as loading controls for the nuclear and cytoplasmic fractions, respectively. **d** IB analysis of the indicated proteins in LATS1/2-wild-type and LATS1/2-null HEK293A and MCF-7 cells after MAP3K3 knockdown. **e** q-RT‒PCR analysis of the expression of MAP3K3 and YAP target genes after MAP3K3 knockdown in LATS1/2-null RPE1 cells (*n* = 3 experiments; ***p < 0.001). **f** IB analysis of CTGF in LATS1/2-null RPE1 and MCF-7 cells after MAP3K3 knockdown. **g** LATS1/2-null RPE1 cells were transfected with the indicated siRNAs and then treated with cycloheximide (100 μg/ml) to inhibit protein synthesis for the indicated times. **h** LATS1/2-null RPE1 cells were transfected with the indicated siRNAs and then treated with DMSO, MG132 (10 μM), concanamycin A (100 nM), NH_4_Cl (10 mM), or bafilomycin A1 (10 nM) for 12 h before harvesting.

Genetic or epigenetic silencing of Hippo signaling is believed to promote YAP activation in the context of cancer, and pharmacological reactivation of Hippo pathway-related gene expression is challenging. We hypothesized that Hippo-independent regulatory mechanisms could serve as viable targets for suppressing YAP activity in cancer. Previously, we performed kinome^5^ and whole-genome siRNA screens^27^ in LATS1/2-null RPE1 cells to identify regulators of YAP levels that operate independently of Hippo kinases. Because the main effectors of the Hippo signaling cascade, LATS1/2, were inactivated in these cell lines, the cells exhibited significantly increased YAP activity^5^, and we also used these cell lines to screen for Hippo-independent YAP regulation pathways. We consistently identified MAP3K3 as one of the top screening hits, and MAP3K3 knockdown was found to significantly reduce the nuclear YAP level. Here, we confirmed this result by YAP immunofluorescence staining in LATS1/2-null RPE1 cells after MAP3K3 depletion via two distinct siRNAs. MAP3K3 knockdown reduced both the nuclear and cytoplasmic YAP immunofluorescence intensities (Fig. 1a). We next tested whether MAP3K3 knockdown reduced the total YAP protein level in LATS1/2 wild-type and LATS1/2-null RPE1 cells. Efficient depletion of MAP3K3 by the siRNAs was confirmed via immunoblot analysis (Fig. 1b). Consistent with the immunofluorescence results, MAP3K3 knockdown reduced the total YAP level independent of the LATS1/2 status (Fig. 1b). Proteasomal degradation of YAP is induced by its phosphorylation at serine 397^10^. However, MAP3K3 knockdown decreased the abundance of YAP phosphorylated at serine 397 proportionally to the reduction in the total YAP level, suggesting that a large increase in YAP serine 397 phosphorylation was not responsible for the decrease in YAP expression induced by MAP3K3 knockdown. Phosphorylation of YAP at serine 127, which mediates its cytoplasmic sequestration, was also proportionally reduced after MAP3K3 knockdown. Immunoblot analysis of subcellular fractions confirmed that MAP3K3 depletion reduced both the nuclear and cytoplasmic YAP levels (Fig. 1c). We also observed a consistent reduction in the total YAP level in HEK293A and MCF-7 cells upon MAP3K3 knockdown, regardless of the LATS1/2 status (Fig. 1d). This reduction was accompanied by a decrease in the level of YAP phosphorylated at serine 127 (Fig. 1d). Taken together, these results suggest that MAP3K3 is required to maintain YAP levels in a LATS1/2-independent manner.

The level of transcription adaptor putative zinc finger (TAZ) was not consistently reduced after MAP3K3 knockdown in RPE1 cells (Fig. 1b). In HEK293T cells, TAZ expression was significantly increased upon LATS1/2 knockout (Fig. 1d). However, the TAZ protein level was not affected by MAP3K3 depletion. In contrast, in MCF-7 cells, LATS1/2 knockout had no impact on TAZ expression, but TAZ expression increased in response to MAPK3K3 knockdown (Fig. 1d). This finding is consistent with a previous report that showed that YAP suppressed TAZ expression^33^. These observations suggest that MAP3K3 does not play a key role in regulating the TAZ protein level and that TAZ expression is strongly influenced by the cellular context.

To test whether MAP3K3 knockdown affects the transcriptional activity of YAP, we examined the expression of the YAP target genes *ANKRD1*, *CTGF*, and *CYR61* after MAP3K3 knockdown. The mRNA levels of these target genes in LATS1/2-null REP1 cells, as well as the CTGF protein level in LATS1/2-null RPE1 and MCF-7 cells, were significantly decreased after MAP3K3 knockdown (Fig. 1e, f), thus confirming the importance of MAP3K3 in the regulation of YAP activity.

The total cellular YAP protein level is determined primarily by the regulation of YAP degradation, which involves both proteasomes and lysosomes^10,34–36^. To test whether MAP3K3 knockdown accelerates YAP degradation, we conducted a cycloheximide chase assay followed by immunoblot analysis of YAP. As shown in Fig. 1g, MAP3K3 knockdown reduced YAP stability in LATS1/2-null RPE1 cells. Furthermore, compared with DMSO control treatment, treatment of MAP3K3-depleted cells with the lysosome inhibitors concanamycin A (CMA), NH_4_Cl, and bafilomycin A1 (BafA1) noticeably increased YAP levels (Fig. 1h). In contrast, the decrease in YAP expression in MAP3K3-depleted cells was only slightly reversed by treatment with the proteasome inhibitor MG132. These findings suggest that MAP3K3 protects YAP primarily from lysosomal degradation.

### MAP3K3 phosphorylates YAP at serine 405

**Fig. 2:**
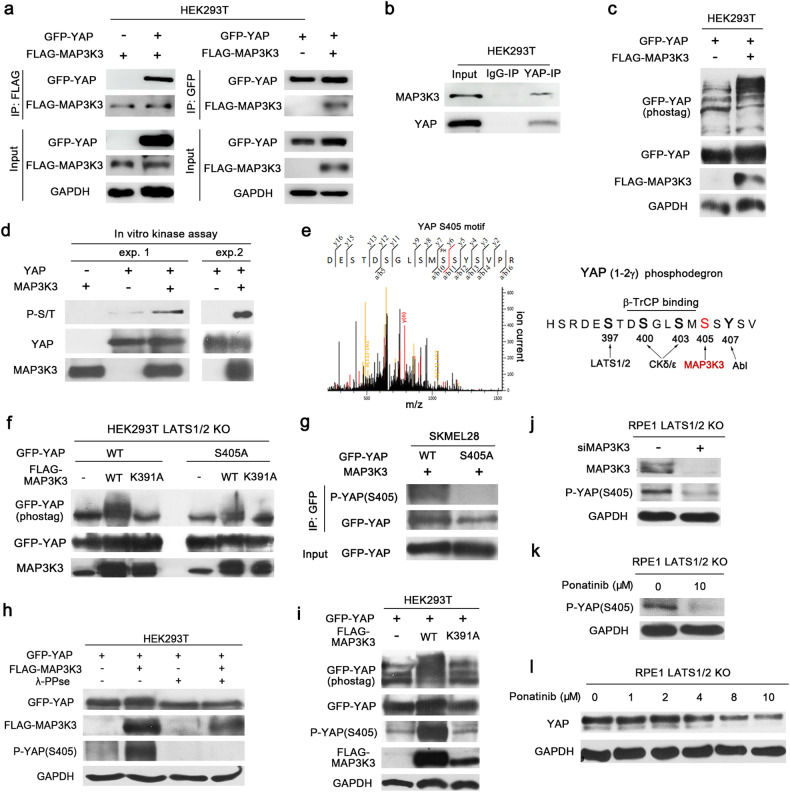
MAP3K3 phosphorylates YAP at serine 405. **a** Reciprocal coimmunoprecipitation (co-IP) assay of lysates from HEK293T cells transfected with GFP-YAP and FLAG-MAP3K3. **b** Co-IP of endogenous YAP and MAP3K3 in HEK293T cells. **c** IB analysis of the indicated proteins in HEK293T cells transfected with GFP-YAP and FLAG-MAP3K3. A Phos-tag-conjugated acrylamide gel was used to detect mobility shifts occurring due to phosphorylation. **d** In vitro kinase assay using purified YAP and MAP3K3 proteins. Phosphorylation was evaluated by immunoblotting using an anti-phospho-serine/threonine antibody. The results from two independent experiments are shown. **e** Mass spectrometry analysis (left) identified the serine 405 residue of YAP as a putative site for MAP3K3-mediated phosphorylation. The illustration (right) indicates the YAP protein sequence surrounding serine 405 and the positions of the phosphorylation sites. Kinases responsible for phosphorylation at each site are also shown. **f** IB analysis of GFP-YAP in LATS1/2-null HEK293T cells transfected with a GFP-conjugated wild-type or S405A mutant YAP vector. The FLAG-tagged wild-type or kinase-dead mutant MAP3K3 (MAP3K3_K391A) vector was also transfected as indicated. A Phos-tag-conjugated acrylamide gel was used to detect mobility shifts occurring due to phosphorylation. **g** IB analysis of phospho-YAP (S405) after immunoprecipitation of GFP-YAP from SKMEL28 cells transfected with MAP3K3 and either wild-type or S405A mutant GFP-YAP. Anti-P-YAP (S405) polyclonal antibodies were generated as part of this study. **h** IB analysis of the indicated proteins in HEK293T cells transfected with GFP-YAP and FLAG-MAP3K3. The lysates were treated with lambda phosphatase as indicated. **i** IB analysis of the indicated proteins in HEK293T cells transfected with GFP-YAP and wild-type or kinase-dead mutant FLAG-MAP3K3. **j** IB analysis of YAP phosphorylated at serine 405 after MAP3K3 knockdown in LATS1/2-null RPE1 cells. **k** IB analysis of phospho-YAP (serine 405) in LATS1/2-null RPE1 cells after treatment with ponatinib for 24 h. **l** IB analysis of YAP in LATS1/2-null RPE1 cells after treatment with ponatinib at the indicated concentrations for 24 h.

A previous study showed that MAP3K3 interacts with YAP through its PPXY motif in C2C12 myoblasts^37^. To test the interaction between MAP3K3 and YAP, we conducted coimmunoprecipitation experiments using exogenously expressed GFP-YAP and FLAG-MAP3K3. Immunoprecipitation using anti-FLAG and anti-GFP antibodies resulted in coprecipitation of GFP-YAP and FLAG-MAP3K3, respectively (Fig. 2a). The reciprocal coimmunoprecipitation experiment confirmed the physical interaction between MAP3K3 and YAP. Coimmunoprecipitation of endogenous YAP and MAP3K3 further demonstrated their physical interaction (Fig. 2b).

To test whether the MAP3K3-YAP interaction leads to YAP phosphorylation, we employed a Phos-tag gel shift assay. Overexpression of FLAG-MAP3K3 in HEK293T cells caused a mobility shift of GFP-YAP in a gel containing Phos-tag acrylamide, indicating that MAP3K3 contributes to YAP phosphorylation (Fig. 2c). We performed an in vitro kinase assay to test whether MAP3K3 directly phosphorylates YAP and observed that purified MAP3K3 phosphorylated YAP at serine/threonine residues in vitro (Fig. 2d).

To identify the specific phosphorylation sites on YAP, we conducted mass spectrometry analysis of GFP-YAP immunoprecipitated from lysates of HEK293T cells overexpressing wild-type or kinase-dead MAP3K3 (MAP3K3_K391A). The analysis identified several residues, including serine 405, that were phosphorylated only in cells overexpressing wild-type MAP3K3 (Fig. 2e). We focused on phosphorylation of serine 405 because this residue is located in the phosphodegron of YAP and is critical for regulating YAP stability^10,38^. We examined whether MAP3K3 can phosphorylate the YAP-S405A mutant to validate serine 405 of YAP as a target site for MAP3K3-mediated phosphorylation. To exclude indirect effects occurring through LATS1/2, we used LATS1/2-null HEK293T cells. Overexpression of MAP3K3 induced a mobility shift in GFP-tagged wild-type YAP in a Phos-tag gel, whereas overexpression of kinase-dead MAP3K3 did not cause a mobility shift in GFP-YAP (Fig. 2f). Moreover, the extent of the MAP3K3-mediated GFP-YAP mobility shift was reduced by S405A mutation of YAP (Fig. 2f). These results indicate that YAP serine 405 is a major target site for MAP3K3-mediated phosphorylation.

We developed a polyclonal antibody targeting YAP phosphorylated at serine 405 [anti-P-YAP (S405)]. The YAP-S405A protein was not recognized by the anti-P-YAP (S405) antibody, confirming the specificity of the antibody (Fig. 2g). Additionally, treatment of lysates with λ-phosphatase resulted in clearance of the anti-P-YAP (S405) antibody-associated immunoblot bands, confirming the phospho-specific activity of the antibody (Fig. 2h). Importantly, the binding of the anti-P-YAP (S405) antibody to GFP-YAP was substantially increased in cells overexpressing wild-type MAP3K3 but not in cells overexpressing kinase-dead MAP3K3 (Fig. 2i). Next, we examined whether a reduction in MAP3K3 expression or activity causes a decrease in YAP phosphorylation at serine 405. MAP3K3 knockdown in LATS1/2 null RPE1 cells resulted in reduced phosphorylation of YAP at serine 405 (Fig. 2j). Additionally, we examined the impact of ponatinib (AP24534), a pan-BCR-ABL kinase inhibitor approved for treating chronic myeloid leukemia and Philadelphia chromosome–positive acute lymphoblastic leukemia. Ponatinib is a multitargeted tyrosine kinase inhibitor, and a previous study showed that ponatinib can effectively inhibit MAP3K3 (MEKK3)-KLF signaling to prevent the progression of cerebral cavernous malformations^39^. Notably, treatment of cells with ponatinib led to decreased YAP phosphorylation at serine 405 (Fig. 2k). Furthermore, ponatinib dose-dependently reduced the total YAP levels (Fig. 2l). Taken together, these findings indicate that MAP3K3 phosphorylates YAP at serine 405.

### Phosphorylation of YAP at serine 405 affects its interaction with E3 ubiquitin ligases

**Fig. 3:**
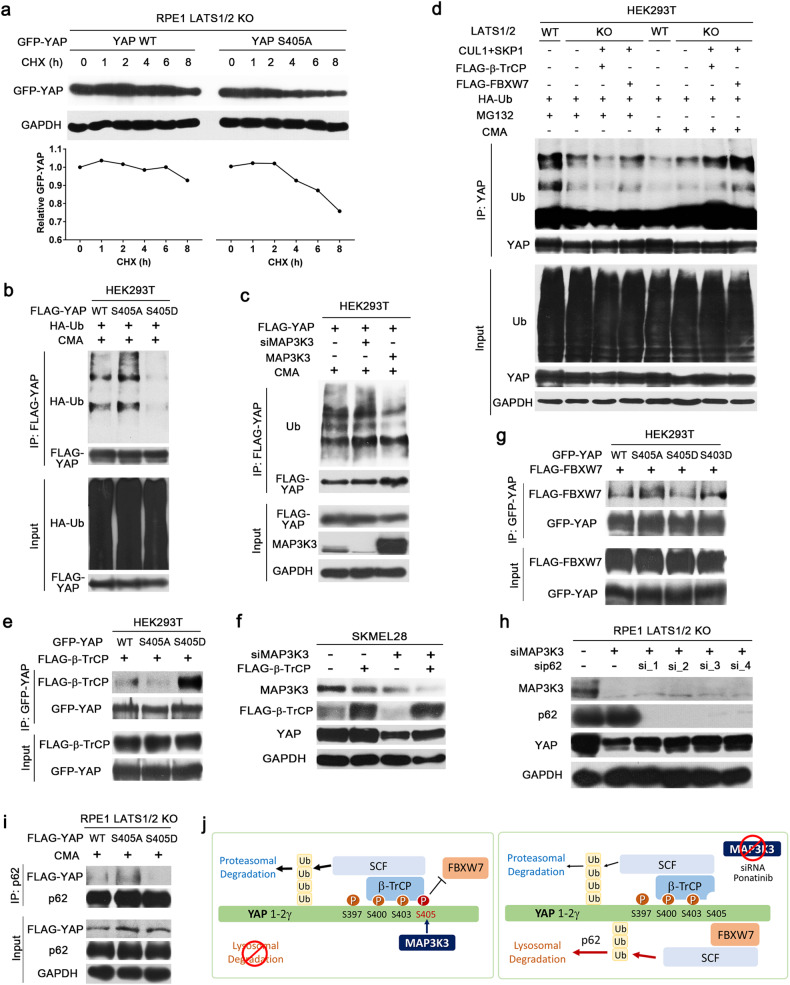
Phosphorylation of YAP at serine 405 influences its ubiquitination and interaction with SCF E3 ubiquitin ligase complex components. **a** IB analysis of exogenous YAP in LATS1/2-null RPE1 cells transfected with the wild-type or S405A mutant YAP vector. Cells were treated with cycloheximide (100 μg/ml) to inhibit protein synthesis for the indicated times before lysis. **b** In vivo ubiquitination assay of YAP immunoprecipitated from HEK293T cells transfected with wild-type or serine 405 mutant YAP and HA-Ubiquitin vectors as indicated. Cells were treated with CMA (100 nM) for 12 h before lysis. **c** Ubiquitination assay of FLAG-YAP immunoprecipitated from HEK293T cells transfected with MAP3K3 siRNA or the MAP3K3 expression vector. Cells were treated with CMA (100 nM) for 12 h before lysis. **d** Ubiquitination assay of YAP immunoprecipitated from LATS1/2-wild-type and LATS1/2-null HEK293A cells transfected with the indicated constructs. The cells were treated with MG132 (10 μM) or concanamycin A (CMA; 100 nM) for 12 h before lysis. **e** Co-IP assay of the interaction between FLAG-β-TrCP and wild-type or serine 405 mutant YAP. **f** IB analysis of YAP in SKMEL28 cells transfected with MAP3K3 siRNA or the FLAG- β-TrCP expression vector as indicated. **g** Co-IP assay of FLAG-FBXW7 and wild-type, serine 405 mutant, or serine 403 mutant YAP. **h** IB analysis of the indicated proteins in LATS1/2-null RPE1 cells transfected with MAP3K3 siRNA or p62 siRNA as indicated. **i** Co-IP assay of p62 and wild-type or serine 405 mutant YAP. The cells were treated with CMA (100 nM) for 12 h before lysis. **j** A speculative model of the mechanism by which MAP3K3 controls YAP degradation.

Next, we investigated whether YAP phosphorylation at serine 405 is required for YAP stability. The cycloheximide chase assay revealed that the stability of the phosphorylation-defective mutant YAP-S405A was lower than that of wild-type YAP (Fig. 3a). To test whether phosphorylation of YAP at serine 405 affects its polyubiquitination, we compared the level of ubiquitin-conjugated YAP under CMA treatment conditions. Compared with wild-type YAP, YAP-S405A exhibited increased polyubiquitination, whereas YAP-S405D, a phosphomimetic mutant, exhibited markedly decreased polyubiquitination (Fig. 3b). Moreover, MAP3K3 overexpression decreased the level of endogenous polyubiquitin-conjugated YAP, while MAP3K3 knockdown slightly increased the level of polyubiquitin-conjugated YAP (Fig. 3c). These results suggest that MAP3K3-mediated phosphorylation of YAP at serine 405 reduces its polyubiquitination and subsequent lysosomal degradation.

Previous studies have demonstrated that both β-TrCP and FBXW7, substrate recognition subunits of the SCF E3 ligase complex, are essential for YAP degradation^10,40^. LATS promotes YAP proteasomal degradation through SCF/β-TrCP-mediated polyubiquitination^10^. Consistent with these findings, the level of polyubiquitinated YAP was lower in the LATS1/2-null cells treated with the proteasome inhibitor MG132 than in the corresponding LATS wild-type cells (Fig. 3d: 1st and 2nd lanes). In addition, the overexpression of SCF E3 ligase complex components in LATS1/2-null cells did not significantly increase the abundance of polyubiquitinated YAP destined for proteasomal degradation (Fig. 3d: 3rd and 4th lanes). LATS1/2-null and wild-type cells treated with CMA to block lysosomal degradation displayed lower levels of polyubiquitinated YAP than did LATS wild-type cells treated with MG132, suggesting that proteasomal degradation predominates under these conditions (Fig. 3d: 1st, 5th and 6th lanes). Interestingly, overexpression of SCF E3 ligase complex components, especially FBXW7, significantly increased the level of polyubiquitinated YAP in LATS1/2-null cells treated with CMA (Fig. 3d: 7th and 8th lanes). These findings suggest that YAP degradation can occur in the absence of Hippo signaling through polyubiquitination by the SCF E3 ligase complex and lysosomal degradation.

We next investigated whether phosphorylation at serine 405 could influence YAP ubiquitination by altering its interaction with substrate recognition subunits of the SCF E3 ligase complex. YAP-S405D exhibited a stronger interaction with β-TrCP than did wild-type YAP, while YAP-S405A showed a slightly weaker interaction with β-TrCP (Fig. 3e). However, the finding that YAP-S405D exhibited a lower level of polyubiquitination in CMA-treated cells (Fig. 3b) suggested that a stronger physical interaction between YAP and β-TrCP may not be sufficient to lead to an increase in polyubiquitination associated with lysosomal degradation. Furthermore, overexpression of β-TrCP did not result in reduced YAP levels (Fig. 3f). Importantly, YAP-S405A exhibited a stronger interaction with FBXW7 than did wild-type YAP (Fig. 3g), suggesting that serine 405 phosphorylation inhibits FBXW7 binding. The interaction of FBXW7 with YAP harboring a phosphomimetic mutation at serine 405 or serine 403, a site for CKδ/ε-mediated phosphorylation^10^, was weaker than that of FBXW7 with YAP-S405A (Fig. 3g). These findings indicate that the phosphorylation status of serine 405 influences the interaction between YAP and the substrate recognition subunits of the SCF E3 ubiquitin ligase complex.

A recent study showed that YAP is an autophagy substrate^34–36^. The autophagy adapter p62 binds to ubiquitinated proteins through its C-terminal ubiquitin-associated domain and promotes autophagosome formation through its LC3-interacting domain^41^. We tested whether YAP phosphorylation by MAP3K3 protects YAP from p62-mediated selective lysosomal degradation. Depletion of p62 reversed YAP downregulation upon MAP3K3 knockdown (Fig. 3h). We also observed that the interaction between p62 and YAP was strengthened due to the S405A mutation but attenuated due to the S405D mutation (Fig. 3i). These results suggest that MAP3K3 controls the YAP-p62 interaction and autophagy/lysosome-mediated YAP degradation.

An overall schematic representation of the mechanism of MAP3K3-mediated regulation of YAP degradation is provided in Fig. 3j. We speculate that dephosphorylation of serine 405 resulting from MAP3K3 inhibition may promote the lysosomal degradation of YAP by enhancing its interaction with FBXW7 and diminishing its interaction with β-TrCP. This, in turn, may lead to alterations in the ubiquitination chain type and the dominant degradation pathway.

### Inhibition of MAP3K3 suppresses YAP activity and overcomes CDK4/6 inhibitor resistance in luminal breast cancer cells

**Fig. 4:**
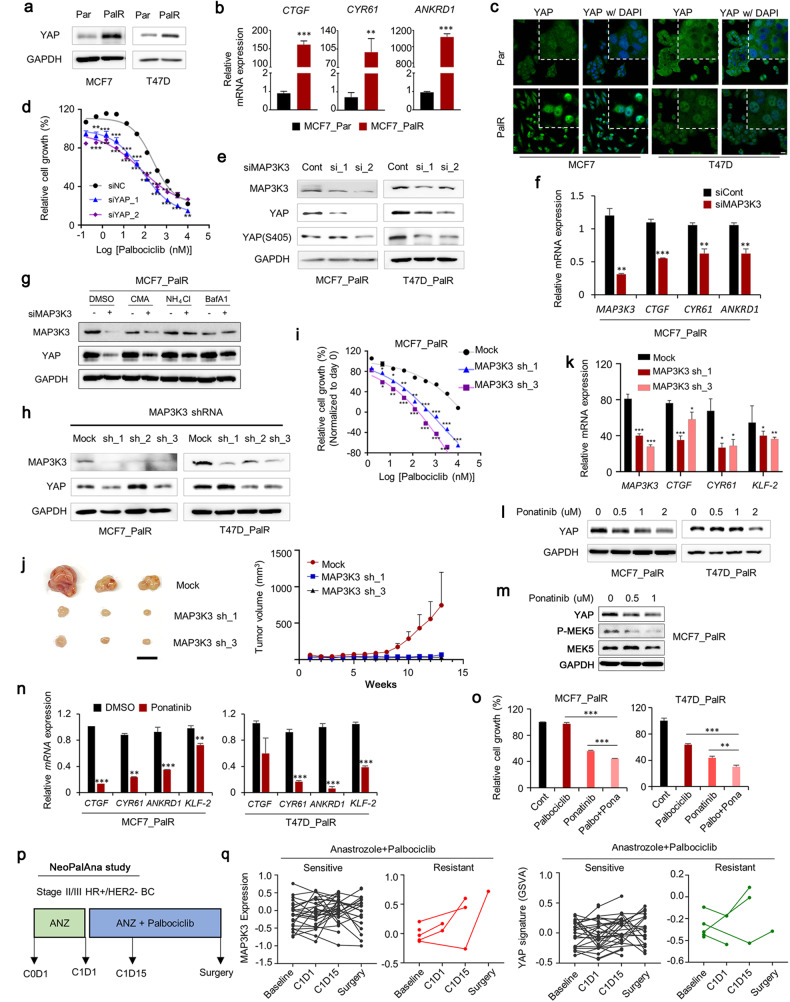
MAP3K3 inhibition counteracts YAP-mediated palbociclib resistance in breast cancer cells. **a** IB analysis of endogenous YAP in parental and palbociclib-resistant MCF7 (MCF7_PalR) and T47D (T47D_PalR) cells. **b** q-RT‒PCR analysis of the expression of YAP target genes in parental and MCF7_PalR cells (*n* = 3 experiments; ***p < 0.001). **c** IF images showing the increased nuclear and cytoplasmic YAP levels in MCF7_PalR and T47D_PalR cells. Scale bar, 20 μm. **d** Palbociclib dose–response curve of MCF7_PalR cells transfected with YAP siRNA. The number of viable cells three days after drug treatment was determined by an MTT assay in 96-well plates. (*n* = 3, ***p < 0.001). **e** IB analysis of the indicated proteins in MCF7_PalR and T47D_PalR cells transfected with MAP3K3 siRNA. **f** q-RT‒PCR analysis of the expression of the MAP3K3 gene and YAP target genes after MAP3K3 knockdown in MCF7_PalR cells (*n* = 3 experiments; ***p < 0.001). **g** Cells were transfected with the indicated siRNAs and treated with DMSO, concanamycin A (100 nM), NH_4_Cl (10 mM), or bafilomycin A1 (10 nM) for 12 h. **h** IB analysis of the indicated proteins in MCF7_PalR and T47D_PalR cells transduced with MAP3K3 shRNA. **i** Palbociclib dose–response curve of MCF7_PalR cells transduced with empty vector or one of the MAP3K3 shRNA vectors. The number of viable cells three days after drug treatment was determined by an MTT assay in 96-well plates (*n* = 3, ***p < 0.001). **j** Xenograft assay using MCF7_PalR cells transduced with empty vector or one of the MAP3K3 shRNA vectors. Photograph showing tumors excised from three mice injected with each cell type 13 weeks after injection. Tumor volume was calculated as 0.5 × L × W^2^, with L indicating the tumor length and W indicating the tumor width. **k** qRT‒PCR analysis of the indicated genes in excised xenograft tumors formed by MCF7_PalR cells transduced with empty vector or one of the MAP3K3 shRNA vectors. **l** Cells were treated with ponatinib at the indicated concentration for 24 h. IB analysis of the indicated proteins was performed. **m** MCF7_PalR cells were treated with ponatinib for 24 h. IB analysis of the indicated proteins was performed. **n** q-RT‒PCR analysis of the indicated genes after ponatinib treatment for 24 h (*n* = 3 experiments; ***p < 0.001). **o** Cells were treated with palbociclib (1 µM) and/or ponatinib (1 µM) for three days. The number of viable cells was determined by an MTT assay in 96-well plates (*n* = 6, ***p < 0.001). **p** The NeoPalAna study design. **q** Graph showing changes in MAP3K3 mRNA expression and YAP signature (YAP1_UP in MSigDB C6) expression according to GSVA in the four groups (baseline, C1D1, C1D15, and surgery). The dataset was obtained from the Gene Expression Omnibus (NeoPalAna trial, GSE93204).

A previous study reported that FAT1 loss in breast cancer cells induced YAP activation, promoting CDK4/6 inhibitor resistance^42^. To investigate whether MAP3K3 inhibition suppresses YAP activity in breast cancer cells resistant to the CDK4/6 inhibitor palbociclib, we established palbociclib-resistant MCF7 (MCF7_PalR) and T47D (T47D_PalR) cell lines by chronic drug administration. Palbociclib is a selective inhibitor of the cyclin-dependent kinases CDK4 and CDK6, and in the landmark phase III clinical trials PALOMA-3^43^ and PALOMA-2^44^, it exhibited a significant progression-free survival benefit in patients with advanced HR-positive and HER2-negative breast cancer. Palbociclib is currently used as a mainstay 1st-line therapy. We observed upregulation of YAP in MCF7_PalR and T47D_PalR cells compared to the corresponding parental cells (Fig. 4a). The expression of the YAP target genes *CTGF*, *CYR61*, and *ANKRD1* was also markedly increased in the resistant cells compared to the parental cells (Fig. 4b). Moreover, the resistant cells exhibited increased nuclear localization of YAP (Fig. 4c). Knockdown of YAP significantly restored sensitivity to palbociclib in MCF7_PalR cells, suggesting that YAP activation is critical for palbociclib resistance (Fig. 4d). Importantly, MAP3K3 knockdown reduced YAP expression in palbociclib-resistant breast cancer cells (Fig. 4e) and downregulated the expression of the YAP target genes *CTGF*, *CYR61*, and *ANKRD1* (Fig. 4f). Treatment of cells with lysosome inhibitors alleviated the reductions in the YAP levels resulting from MAP3K3 knockdown, indicating that lysosomal degradation of YAP occurred in cells depleted of MAP3K3 (Fig. 4g).

Next, to conduct xenograft experiments, we established resistant cells stably expressing MAP3K3 shRNAs (Fig. 4h). There was some heterogeneity in the cellular response to MAP3K3 shRNA transduction, possibly due to changes in cell characteristics during the selection process after transduction. We selected two MCF7_PalR cell lines (sh_1 and sh_3) for the xenograft study. We observed significantly greater sensitivity to palbociclib in MCF7_PalR cells harboring MAP3K3 shRNAs than in mock-transduced MCF7_PalR cells (Fig. 4i). Compared with the corresponding control MCF7_PalR cells, injected MCF7_PalR cells expressing MAP3K3 shRNAs exhibited a significantly decreased capacity to form tumors (Fig. 4j). YAP target genes were also downregulated in tumors formed by MCF7_PalR cells expressing MAP3K3 shRNAs (Fig. 4k). These results demonstrate that YAP-dependent proliferation of breast cancer cells in vivo can be suppressed by depletion of MAP3K3.

Notably, we observed a dose-dependent reduction in the YAP protein level in MCF7_PalR and T47D_PalR cells in response to ponatinib treatment (Fig. 4l). The inhibition of MAP3K3 by ponatinib was confirmed by the reduction in the level of phosphorylated MEK5, the substrate of MAP3K3 (Fig. 4m). Furthermore, the expression of YAP target genes in resistant cells was reduced by ponatinib (Fig. 4n). Combination treatment with ponatinib and palbociclib significantly reduced the viability of the resistant cells, demonstrating that pharmacological MAP3K3 inhibition suppresses YAP-mediated CDK4/6 inhibitor resistance (Fig. 4o).

We analyzed mRNA expression data from the NeoPalAna trial (GSE93204)^30^ to assess the associations among MAP3K3 expression, the YAP signature, and drug resistance in patients with luminal breast cancer (Fig. 4p). In this trial, patients with estrogen receptor-positive breast cancer were initially treated with anastrozole for 28 days and were then transitioned to anastrozole + palbociclib treatment. Tumor biopsies were taken at baseline (Cycle 0 Day1 [C0D1]), at the start of anastrozole + palbociclib treatment (C1D1), at 15 days into the combined treatment phase (C1D15), and at surgery (after 4-5 months of the combined treatment). We compared changes in MAP3K3 RNA expression and the YAP signature between two groups of patients: patients who were sensitive to anastrozole + palbociclib (*n* = 34, Ki67 <= 2.7% at C1D15 biopsy) and patients who were resistant to this combination treatment (*n* = 5, Ki67 > 2.7% at C1D15 biopsy). The baseline MAP3K3 expression level did not significantly differ between the sensitive and the resistant patients. However, following anastrozole + palbociclib treatment, the MAP3K3 and YAP signature expression levels appeared to increase in resistant patients but not in sensitive patients (Fig. 4q). These findings suggest that MAP3K3 upregulation and YAP activation may play a role in clinical resistance to palbociclib treatment in patients with luminal breast cancer.

### Elevated MAP3K3 protein expression is associated with YAP upregulation in BRAF inhibitor-resistant melanoma cells

**Fig. 5:**
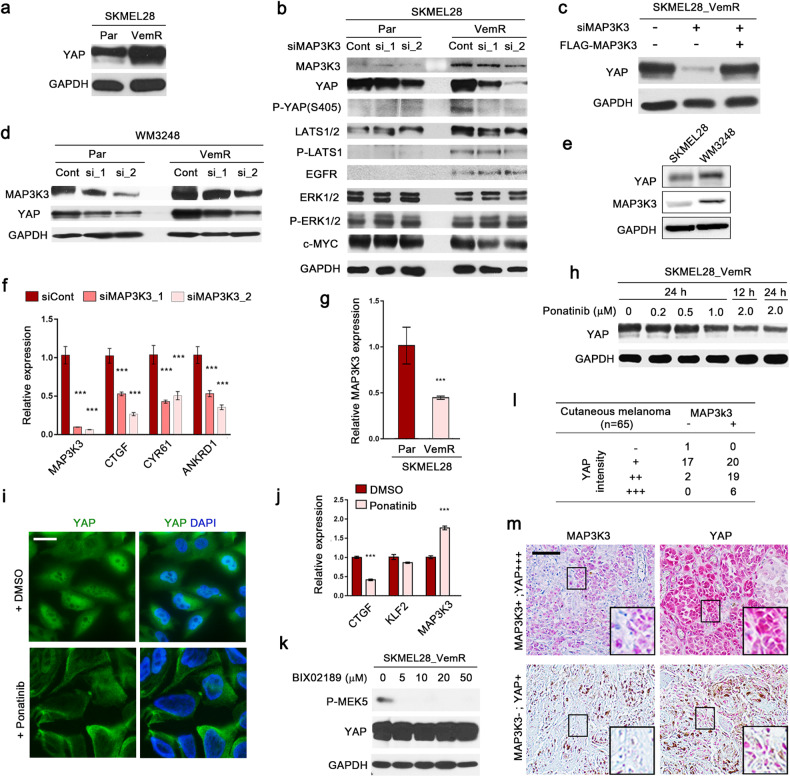
MAP3K3 upregulates YAP in BRAF inhibitor-resistant melanoma cells. **a** IB analysis of the indicated proteins in parental and BRAF inhibitor-resistant (VemR) SKMEL28 melanoma cells. **b** IB analysis of the indicated proteins in parental and SKMEL28_VemR cells transfected with MAP3K3 siRNAs. **c** IB analysis of YAP in SKMEL28_VemR cells transfected with MAP3K3 siRNA and a siRNA-insensitive MAP3K3 expression vector. **d** IB analysis of the indicated proteins in parental and WM3248_VemR cells transfected with MAP3K3 siRNAs. **e** IB analysis of the indicated proteins in parental SKMEL28 and WM3248 cells. **f** q-RT‒PCR analysis of the expression of YAP target genes after MAP3K3 knockdown in SKMEL28_VemR cells (*n* = 3 experiments; ***p < 0.001). **g** q-RT‒PCR analysis of MAP3K3 expression in parental and SKMEL28_VemR cells (*n* = 3 experiments; ***p < 0.001). **h** IB analysis of YAP in SKMEL28_VemR cells treated with ponatinib at the indicated concentration for 12 or 24 h. **i** IF micrographs show**i**ng YAP downregulation in VemR SKMEL28 cells treated with ponatinib (1 μM) for 24 h. Scale bar, 20 μm. **j** q-RT‒PCR analysis of the indicated genes in SKMEL28_VemR cells treated with ponatinib (1 μM) for 24 h (*n* = 3 experiments; ***p < 0.001). **k** IB analysis of the indicated proteins in SKMEL28_VemR cells treated with BIX02189 at the indicated concentration for 24 h. **l** Classification of 65 human cutaneous melanoma samples based on the expression of MAP3K3 and YAP. **m** Representative images of MAP3K3 and YAP IHC staining in human cutaneous melanoma tissues. Scale bar, 200 μm. Red represents immunostaining, and brown indicates melanosome pigment.

We previously reported that melanoma cells resistant to the BRAF inhibitor vemurafenib (PLX4032) exhibited increased actin stress fiber formation, which promoted YAP nuclear accumulation^28^. Consistently, the total YAP level was increased in SKMEL28 cells resistant to vemurafenib (SKMEL28_VemR) compared to the corresponding parental cells (Fig. 5a). Notably, we observed an increase in the MAP3K3 level in SKMEL28_VemR cells, which was consistent with the increase in YAP phosphorylation at serine 405 (Fig. 5b). MAP3K3 depletion in SKMEL28_VemR cells resulted in decreased levels of total YAP and YAP phosphorylated at serine 405, while the level of total YAP was only slightly reduced in the parental cells (Fig. 5b). Consistent with the findings of a previous report^28^, YAP downregulation in resistant cells was accompanied by a reduction in the c-MYC level, while no significant changes in ERK1/2 phosphorylation were induced by MAP3K3 knockdown (Fig. 5b). Expression of the RNAi-insensitive MAP3K3 construct efficiently reversed the downregulation of YAP caused by MAP3K3 siRNA transfection (Fig. 5c). Another melanoma cell line, WM3248, also presented increased MAP3K3 and YAP levels after the acquisition of vemurafenib resistance (Fig. 5d). Furthermore, MAP3K3 knockdown resulted in a reduction in YAP expression in vemurafenib-resistant WM3248 cells (Fig. 5d). The parental WM3248 cells showed higher expression of MAP3K3 than did the parental SKMEL28 cells (Fig. 5e). Consistent with this finding, MAP3K3 knockdown caused a reduction in YAP expression in parental WM3248 cells (Fig. 5d). MAP3K3 knockdown in SKMEL28_VemR cells resulted in decreased expression of the YAP target genes *CTGF*, *CYR61*, and *ANKRD1* (Fig. 5f). These results suggest that MAP3K3 upregulation during the acquisition of drug resistance contributes to increases in YAP levels. We tested whether transcriptional upregulation of *MAP3K3* is responsible for the increase in the MAP3K3 protein level in resistant cells. Unexpectedly, we observed a reduction in *MAP3K3* transcription in SKMEL28_VemR cells, which suggested the existence of a posttranscriptional/posttranslational mechanism associated with MAP3K3 upregulation (Fig. 5g).

Next, we examined the effect of ponatinib-induced MAP3K3 inhibition on vemurafenib-resistant SKMEL28 cells. Immunoblot analysis indicated that ponatinib treatment decreased YAP levels in vemurafenib-resistant cells in a dose-dependent manner (Fig. 5h). Immunofluorescence staining using an anti-YAP antibody also demonstrated a reduced YAP level in ponatinib-treated cells (Fig. 5i). Ponatinib inhibited the transcription of *CTGF*, whereas the expression of *KLF-2*, a target of MEK5/ERK5 signaling, was unaffected (Fig. 5j). The transcription of *MAP3K3* increased in response to ponatinib treatment, possibly due to the attenuation of negative feedback regulation (Fig. 5j). These results further support the idea that MAP3K3 inhibition is an efficient method for suppressing YAP expression in vemurafenib-resistant melanoma cells. To rule out the possibility that ponatinib-induced YAP downregulation occurred due to inhibition of the MEK5/ERK5 pathway, we tested the impact of the MEK5 inhibitor BIX02189 on the YAP levels. As shown in Fig. 5k, treatment with the MEK5 inhibitor did not noticeably alter the YAP levels.

Several reports have provided in vitro and in vivo evidence that increased YAP activity is associated with the malignancy of cutaneous melanoma^45,46^. To further demonstrate the potential role of MAP3K3 in stabilizing YAP in the context of cancer, we investigated the association between MAP3K3 and YAP expression in malignant cutaneous melanoma samples. Immunohistochemical staining for MAP3K3 and YAP was performed on specimens from 65 patients with cutaneous melanoma. When we reviewed the expression levels of MAP3K3 and YAP, melanomas with MAP3K3 expression had higher expression levels of YAP than did melanomas without MAP3K3 expression (*p* = 0.0039; Fig. 5l, m).

### MAP3K3 contributes to YAP-dependent BRAF inhibitor resistance and malignant behavior in melanoma cells

**Fig. 6:**
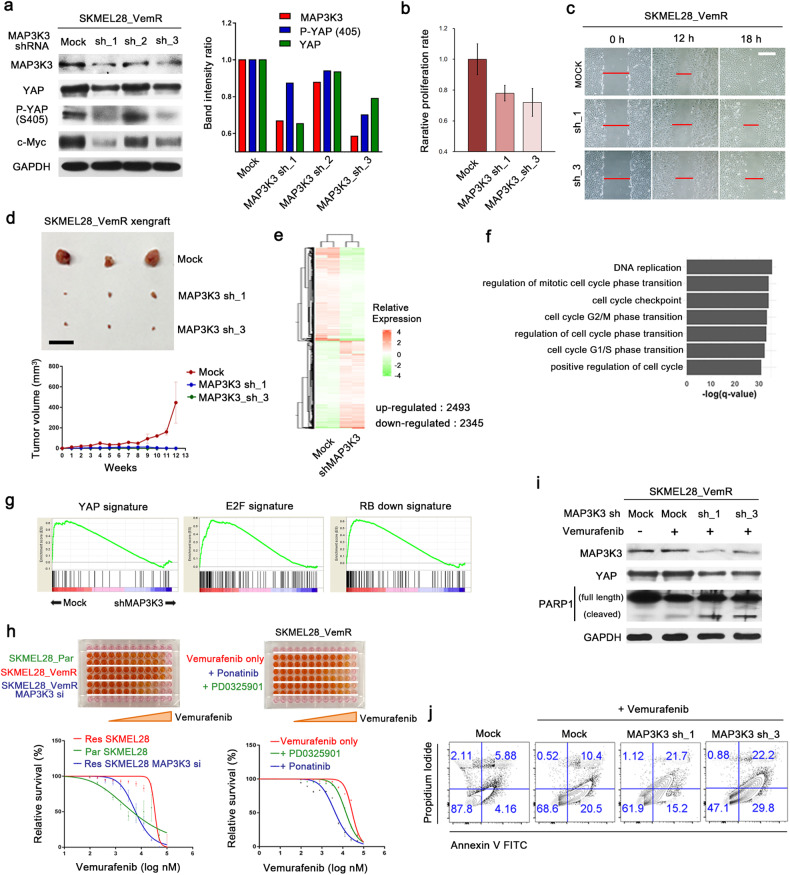
MAP3K3 inhibition reduces melanoma cell proliferation and BRAF inhibitor resistance. **a** IB analysis of the indicated proteins in SKMEL28_VemR cells transduced with empty vector or a MAP3K3 shRNA vector (left). The bar graph (right) shows the band intensity ratios between the samples. The band intensities for each sample were normalized to the band intensity of GAPDH. **b** Proliferation assay of SKMEL28_VemR cells transduced with empty vector or one of the MAP3K3 shRNA vectors. Cells were seeded at 0.5 × 10^4^ cells per well in 96-well plates, and the cell density was quantified using a Cell Counting Kit-8 (CCK-8) after three days. **c** Scratch assay of SKMEL28_VemR cells transduced with empty vector or a MAP3K3 shRNA vector. Scale bar, 200 μm. **d** Xenograft assay using SKMEL28_VemR cells transduced with empty vector or one of the MAP3K3 shRNA vectors. The photograph shows tumors excised from three mice injected with each cell type 13 weeks after injection (upper). Tumor volume was calculated as 0.5 × L × W^2^, with L indicating the tumor length and W indicating the tumor width (bottom). **e** Heatmap showing the expression levels of genes with significantly altered expression in SKMEL28_VemR cells transduced with a MAP3K3 shRNA vector compared to cells transduced with the empty vector (RNA sequencing data; *n* = 2 biological replicates). **f** Gene Ontology analysis of genes significantly downregulated upon MAP3K3 depletion in SKMEL28_VemR cells. **g** GSEA enrichment plots demonstrating the downregulation of YAP, E2F1, and RB loss-of-function signatures after MAP3K3 depletion in SKMEL28_VemR cells. **h** Vemurafenib dose‒response curve of parental and vemurafenib-resistant cells and SKMEL28_VemR cells transfected with MAP3K3 siRNA (left). Vemurafenib dose‒response curve of SKMEL28_VemR cells cultured in the presence of ponatinib or PD0325901 (right). The number of viable cells three days after drug treatment was determined by a CCK-8 assay in 96-well plates. **i** IB analysis of PARP1 cleavage in SKMEL28_VemR cells transduced with empty vector or a MAP3K3 shRNA vector. Cells were treated with vemurafenib (10 μM) for 72 h. **j** Flow cytometric detection of Annexin V- and propidium iodide-positive cells. SKMEL28_VemR cells transduced with the empty vector or a MAP3K3 shRNA vector were treated with vemurafenib (10 μM) for 72 h.

We attempted to generate a vemurafenib-resistant MAP3K3-null SKMEL28 cell line using the CRISPR/Cas9 system. However, the establishment of viable clones was unsuccessful due to the toxic effects of complete loss of MAP3K3. We established vemurafenib-resistant SKMEL28 cells stably expressing MAP3K3 shRNA. We analyzed three clones and found that the reductions in the total YAP and serine 405-phosphorylated YAP levels were proportional to the efficiency of MAP3K3 knockdown (Fig. 6a). The degree of c-Myc downregulation also corresponded to the efficiency of MAP3K3 knockdown (Fig. 6a). To test whether MAP3K3 knockdown can suppress the malignant behavior of vemurafenib-resistant melanoma cells, we examined the cell proliferation and migration ability abilities in vitro and the tumor formation capacity in xenografted immunocompromised mice. Cell proliferation and in vitro scratch assays showed that the proliferation and migration of SKMEL28_VemR cells stably expressing MAP3K3 shRNAs were significantly lower than those of SKMEL28_VemR cells harboring empty shRNA vector (Fig. 6b, d c). Moreover, unlike control SKMEL28_VemR cells, SKMEL28_VemR cells expressing MAP3K3 shRNAs could not form tumors in immunocompromised mice (Fig. 6d).

To evaluate the impact of MAP3K3 knockdown on the transcriptome of melanoma cells, we performed RNA-seq analysis with SKMEL28 cells harboring control or MAP3K3 shRNA. The expression of 2493 genes was upregulated and that of 2345 genes was downregulated in MAP3K3-depleted vemurafenib-resistant cells (cutoff criteria = fold change > 2.0, *q* value < 0.05) (Fig. 6e). Gene Ontology analysis^47^ revealed that cell cycle and mitosis were the functional categories most enriched in the downregulated genes in MAP3K3-depleted cells (Fig. 6f). In addition, GSEA^32^ using data from MSigDB (C6 oncogenic signatures) showed that the gene sets associated with the YAP, E2F, and RB loss-of-function signatures were significantly downregulated upon MAP3K3 knockdown in vemurafenib-resistant SKMEL28 cells (Fig. 6g). These data are consistent with those of previous studies on the effect of YAP/TAZ knockdown in vemurafenib-resistant melanoma cell lines^28^. Taken together, these results suggest that inhibition of MAP3K3 suppresses YAP function in melanoma cells.

Finally, we investigated the potential of MAP3K3 inhibition to counteract vemurafenib resistance. We found that susceptibility to vemurafenib increased when MAP3K3 was inhibited through siRNA transfection or ponatinib treatment (Fig. 6h). While ponatinib may have an impact on MEK/ERK signaling, the combination of vemurafenib and ponatinib more effectively reduced cell survival than did the combination of vemurafenib and the MEK inhibitor PD0325901 (Fig. 6h). This finding suggests that YAP inhibition is the primary mechanism by which ponatinib overcomes BRAF inhibitor resistance. We next examined whether MAP3K3 knockdown increased the levels of cell death markers after vemurafenib treatment. Compared with control SKMEL28_VemR cells, MAP3K3 shRNA-expressing SKMEL28_VemR cells showed higher levels of the apoptosis marker cleaved PARP (Fig. 6i) and late apoptosis/necrosis indicators (Fig. 6j) after vemurafenib treatment. Therefore, we conclude that MAP3K3 contributes to YAP-dependent BRAF inhibitor resistance and other malignant behaviors in melanoma cells and is a promising target for anticancer drug development.

## Discussion

YAP is a potent driver of malignant transformation in cancer cells, and YAP activation via genetic and epigenetic mechanisms is frequently observed in various human cancers^48,49^. The Hippo tumor suppressor pathway plays a major role in restricting YAP activity. However, loss-of-function mutations in core Hippo kinase genes occur relatively infrequently, and genetic depletion of any of several Hippo pathway components does not promote cancer development^46,50^. We suggest that the dysregulation of Hippo-independent YAP regulators may be critical for YAP hyperactivity in cancer. In this study, we identified MAP3K3 as a novel positive regulator of YAP stability that directly phosphorylates YAP. We found that YAP activity is dependent on MAP3K3 in CDK4/6 inhibitor-resistant breast cancer cells and BRAF inhibitor-resistant melanoma cells. Inhibition of MAP3K3 by shRNA transduction or ponatinib treatment suppressed YAP-dependent resistance to the CDK4/6 inhibitor palbociclib, which is a mainstay targeted therapy for hormone-positive HER2-negative breast cancers. Moreover, depletion of MAP3K3 decreased the tumorigenic potential and BRAF inhibitor resistance of melanoma cells. Our findings suggest that MAP3K3 is a promising target for alleviating drug resistance and that combination treatment with ponatinib with inhibitors of BRAF or CDK4/6 should be further tested as a YAP-targeting strategy in cancer treatment.

Osmotic stress, inflammatory mediators, and nutrients trigger MAP3K3-dependent signal transduction^20,51–53^, activating downstream MAPK cascades and NF-κB signaling in a context-dependent manner^21,54^. In addition, MAP3K3 has been shown to regulate autophagic flux by influencing mTOR activity^52^. Elevated MAP3K3 expression is also associated with the progression and prognosis of several cancers, such as esophageal squamous cell carcinoma, cervical cancer, and ovarian cancer^23–25^. Santoro et al. reported that MAP3K3 can sustain pancreatic cancer by supporting the oncogenic activity of YAP and TAZ^26^. They reported significantly decreased expression of YAP target genes in MAP3K3-null pancreatic cancer cells. In the present study, we found that MAP3K3 regulates YAP in RPE1 and HEK293 cells, as well as in melanoma and breast cancer cells, suggesting that MAP3K3-mediated YAP regulation is a general mechanism. However, the role of MAP3K3 in YAP regulation is likely to also be influenced by cellular characteristics, such as the expression levels of alternative YAP regulators or MAP3K3. The impact of MAP3K3 knockdown on YAP protein levels was less pronounced in parental SKMEL28 cells, which expressed lower levels of MAP3K3, than in their vemurafenib-resistant counterparts (Fig. 5b). Notably, LATS1/2 deletion did not affect the MAP3K3 knockdown-mediated decreases in YAP levels (Fig. 1). Therefore, highly expressed MAP3K3 may function as a hub in a complex regulatory circuit for YAP regulation that acts in parallel with Hippo signaling.

MAP kinases are phylogenetically conserved signaling tools that mediate a wide variety of external inputs to ensure proper cellular responses. Therefore, the interaction between the Hippo–YAP pathway and MAP kinases is not an unexpected finding. MAP4Ks are involved in the regulation of LATS1/2 activity^15,16^. The interaction between MAP2K1 (MEK1) and YAP is required for the proliferation of liver cancer cells^17^. MEK1 nuclear localization can sequester β-TrCP from cytoplasmic YAP, thus stabilizing YAP, in KRAS-mutant colon cancer^52,55^. ERK1/2, JNK, and p38 are also positive regulators of YAP in non-small cell lung cancer cells and during alveolar regeneration^18,56^. Since MAP3K3 acts independently of LATS1/2 and directly phosphorylates YAP, the MAP3K3-mediated mechanism of YAP control is distinct from the above MAPK-dependent YAP regulatory pathways. However, the activation of other MAP kinases, NF-κB, or mTOR signaling pathways by MAP3K3 may also modulate the signaling networks involved in YAP activity and stability in certain cellular environments.

We found that MAP3K3 phosphorylates YAP at serine 405, which prevents the polyubiquitination and p62-dependent lysosomal degradation of YAP. Previous studies have indicated that YAP levels are regulated via both proteasomal and lysosomal degradation pathways. At high cell densities, the half-life of the YAP protein is controlled by LATS-mediated phosphorylation and subsequent proteasomal degradation. At low cell densities, the half-life of YAP is controlled by nutrient availability and the mTOR pathway, which suppresses autophagic/lysosomal degradation of YAP^57^. LATS-mediated phosphorylation at serine 397 increases the polyubiquitination of YAP by promoting the interaction between YAP and β-TrCP. Unexpectedly, we observed a stronger physical interaction between β-TrCP and YAP harboring a phosphomimetic mutation at serine 405 (S405D) (Fig. 3e). It is possible that a stronger interaction between β-TrCP and YAP prevents YAP lysosomal degradation, consequently maintaining a certain level of total YAP protein. In line with this, overexpression of β-TrCP alone did not decrease YAP levels (Fig. 3f). YAP-S405A exhibited a decreased interaction with β-TrCP (Fig. 3e) but an increased interaction with FBXW7 (Fig. 3g). Based on these findings, we propose a speculative model for YAP stability regulation (Fig. 3j): (i) β-TrCP-mediated ubiquitination primarily promotes the proteasomal degradation of YAP, while FBXW7-mediated ubiquitination mediates its lysosomal degradation; (ii) YAP phosphorylation at serine 405 by MAP3K3 prevents the YAP-FBXW7 interaction and the lysosomal degradation of YAP; and (iii) serine 405 phosphorylation promotes β-TrCP binding to YAP, further suppressing the lysosomal degradation of YAP. Consistent with this model, a previous study reported that β-TrCP plays a dual role in regulating YAP stability^17^. β-TrCP accelerates YAP turnover when the Hippo pathway is activated, whereas under conditions of low Hippo pathway activity, it protects YAP from degradation. Notably, while FBXW7 is known to be a tumor suppressor, β-TrCP can function as either an oncogene or a tumor suppressor.

Upregulation of YAP activity is associated with the development of BRAF inhibitor resistance^28,58^. In this study, we demonstrated the potential role of MAP3K3 in BRAF inhibitor resistance in melanoma cells. We found that both the nuclear localization and total abundance of YAP were increased in vemurafenib-resistant cells. We surveyed the Cancer Genome Atlas cutaneous melanoma dataset using cBioPortal^59^ and found *MAP3K3* alterations (mutation, amplification, or mRNA upregulation) in 12% of the tumor samples. Treatment with a CDK4/6 inhibitor plus endocrine therapy is now the standard therapeutic regimen for hormone-positive, HER2-negative advanced breast cancer. However, clinically feasible biomarkers for the CDK4/6 inhibitor response have not been developed. Moreover, a previous study reported that the resistance mechanism was unknown in a considerable subset of patients (34%)^60^. In this study, we showed that chronic treatment with palbociclib can promote the development of YAP-dependent drug resistance in breast cancer cells. Epigenetic YAP activation following CDK4/6 inhibitor treatment may be a common occurrence in patients with luminal breast cancer. We also demonstrated that MAP3K3 inhibition can overcome palbociclib resistance. Combining a CDK4/6 inhibitor with ponatinib or other MAP3K3 inhibitors may mitigate drug resistance, leading to an enhanced response and prolonged disease control. However, further in vivo studies are needed to elucidate the importance of MAP3K3 and YAP in cancer progression and drug resistance.

In conclusion, we discovered a novel regulatory mechanism of YAP degradation mediated by MAP3K3, which blocks the YAP-FBXW7 interaction and p62-mediated YAP lysosomal degradation. Furthermore, we demonstrated that pharmacological MAP3K3 inhibition can overcome YAP-induced resistance to CDK4/6 inhibitors in breast cancer cells and to BRAF inhibitors in melanoma cells, underscoring the therapeutic relevance of our findings.

### Supplementary information

Supplemental Material
