# Supplementary material for: Overcoming BRAF and CDK4/6 inhibitor resistance by inhibiting MAP3K3-dependent protection against YAP lysosomal degradation
Source: Exp Mol Med. 2024 Apr 16;56(4):987–1000. doi: 10.1038/s12276-024-01210-5 (PMC11059244; doi:10.1038/s12276-024-01210-5)
Supplement: Supplementary file 1 — Supplemental Material [file 12276_2024_1210_MOESM1_ESM.pdf]

SUPPLEMENTARY MATERIALS AND METHODS

Immunofluorescence and microscopic examination

Cells were seeded onto eight-well Lab-Tek II chamber slides (Nunc) and fixed with 4 % paraformaldehyde for 7 min at room temperature. After fixation, 0.1–0.3 % Triton X-100 (Sigma Aldrich) was used to permeabilize cell membranes for 10 min at room temperature. The cells were then incubated with the primary antibodies for 1 h at room temperature. Bound primary antibodies were detected by incubation with Alexa Fluor 488- or 594-conjugated secondary antibodies for 45 min at room temperature. DAPI was added to the mounting solution to stain the nuclei. Fluorescence images were acquired using a DeltaVision Spectris Imaging System equipped with a cooled CCD camera (Applied Precision).

Cell viability assay

For vemurafenib dose-response analysis, 1×10^4 cells per well were seeded in 96-well plates after siRNA transfection for 24 h. After transfection for 72 h, the cells were treated with variable doses of vemurafenib for 72 h. Viable cells were quantified by measuring the absorbance at 450 nm 2 h after treatment with the Cell Counting Kit-8 reagent (CCK8; Dojindo). For vemurafenib dose-response analysis after drug treatment, cells were incubated for 24 h after plating and treated with the drugs indicated in the figure legends and with variable doses of vemurafenib for 72 h before the CCK8 assay. To confirm the acquisition of palbociclib resistance, MCF7, MCF7_PalR, T47D, and T47D_PalR cells (5×10^3 cells/well) were seeded in 96-well plates. After 24 h, the cells were treated with various doses of palbociclib for three (MCF7 and MCF7_PalR) or five days (T47D and T47D_PalR). Viable cells were quantified by measuring the absorbance at 570 nm after treatment with MTT reagent (5 mg/ml) for 2 h.

Scratch assay

SKMEL28 cells were cultured until they reached confluency. Cell monolayers were scratched with a P200 pipette tip and washed with the medium to remove cell debris. The cells were photographed at 0, 12, and 18 h after scratching.

Tumor xenograft assay

Vemurafenib-resistant SKMEL28 cells or palbociclib-resistant MCF7 cells carrying empty or MAP3K3 shRNA vectors were subcutaneously injected into the right flank of female BALB/c nude

mice (1 × 10^6 cells each for SKMEL28 cells and 1 × 10^7 cells each for MCF7 cells). Tumor size was measured every week using calipers, and the tumor volume was calculated as 0.5 × L × W^2, with L indicating length and W indicating width. The mice were euthanized 13 weeks after injection.

Annexin V apoptosis assay

Vemurafenib-resistant SKMEL28 cells were cultured in the absence or presence of vemurafenib. Apoptotic and dead cells were labeled with annexin V-FITC and stained with propidium iodide, according to the manufacturer's protocol (Invitrogen). An LSR Fortessa flow cytometer (488 nm and 633 nm; BD Biosciences) was used to count the labeled cells. Data were analyzed using FACSDiva software (BD Biosciences).

Immunohistochemistry (IHC) of human cutaneous melanoma tumors

Cutaneous melanoma tissues (n = 65) were retrieved from the Department of Pathology at Severance Hospital, Seoul, Korea. IHC was performed with anti-YAP and anti-MAP3K3 antibodies using an automated immunohistochemical staining instrument (Ventana BenchMark XT; Ventana Medical System), according to the manufacturer's instructions. The Ultraview Universal Alkaline Phosphatase Red Detection Kit (Roche Diagnostics) was used for detection. IHC slides were scored by an expert pathologist (S. Kim) who was blinded to the molecular data. The YAP staining intensity was classified as negative, weakly positive, moderately positive, or strongly positive.

LC-MS/MS analysis

Protein bands in the SDS-PAGE gel stained with colloidal blue were excised and digested with trypsin. Tryptic peptides were extracted using 1% formic acid containing 50% acetonitrile for 20 min with mild sonication. The extracted solution was then concentrated using a centrifugal vacuum concentrator. Prior to mass spectrometric analysis, the peptides solution was subjected to a desalting process using a reversed-phase column. LC-MS/MS analysis was performed using a nano ACQUITY UPLC and LTQ-orbitrap-mass spectrometer (Thermo Electron) with a BEH C18 column (1.7 μm, 100 μm × 100 mm; Waters). For tandem mass spectrometry, mass spectra were acquired via data-dependent acquisition with a full mass scan (300–2000 m/z) followed by MS/MS scans. Each MS/MS scan acquired was the average of one microscan of the LTQ. The temperature of the ion transfer tube was maintained at 275 °C, and the spray voltage was 2.0 kV. The normalized collision energy was set to 35% for MS/MS. The individual spectra from MS/MS

were processed using SEQUEST software (Thermo Quest) and the generated peak lists were used to query the in-house database using the MASCOT program (Matrix Science).

Quantitative reverse transcription-polymerase chain reaction (RT-PCR)

Total RNA was extracted from cells using TRIzol reagent according to the manufacturer's protocol (Invitrogen). A total of 1 μg of extracted RNA was transcribed into cDNA using the High Capacity cDNA Reverse Transcription Kit (Applied Biosystems). To analyze gene expression, real-time PCR was performed using a StepOne Real-Time PCR system and Fast SYBR Green Master Mix (ThermoFisher). Relative quantification was based on the ddCt method, and *36B4* and *GAPDH* genes were used as the internal controls. Primers were adopted from previously published studies or designed using Primer-BLAST.

Supplementary Table 1.

REAGENT OR RESOURCE	SOURCE	IDENFITIER
**Antibodies**		
Anti-YAP (63.7)	Santa Cruz Biotechnology	Cat# sc101199
Anti-phospho-YAP-Ser127	Cell Signaling Technology	Cat# 4911
Anti-phospho-YAP-Ser397 (D1E7Y)	Cell Signaling Technology	Cat# 13619
Anti-phospho-YAP-Ser405	This study	N/A
Anti-TAZ (H-70)	Santa Cruz Biotechnology	Cat# sc48805
Anti-MAP3K3 (ep600y)	Abcam	Cat# ab40756
Anti-LATS1/2	Abcam	Cat# ab70565
Anti-phospho-LATS1-Ser909	Cell Signaling Technology	Cat# 9157
Anti-CTGF (E-5)	Santa Cruz Biotechnology	Cat# sc365970
Anti-c-Myc (Y69)	Abcam	Cat# ab32072
Anti-ERK1/2	Cell Signaling Technology	Cat# 9102
Anti-phospho-ERK1/2-Thr202/Tyr204 (197G2)	Cell Signaling Technology	Cat# 4377
Anti-IL-6	Santa Cruz Biotechnology	Cat# sc130326
Anti-phosphoserine/threonine (22A)	BD bioscience	Cat# 612548
Anti-Ub (P4D1)	Santa Cruz Biotechnology	Cat# sc8017
Anti-β-TrCP (D13F10)	Cell Signaling Technology	Cat# 4394
Anti-p62	MBL	Cat# pm045
Anti-PARP-1 (H250)	Santa Cruz Biotechnology	Cat# sc7150
Anti-GFP	Abcam	Cat# ab290
Anti-FLAG (M2)	Sigma	Cat# F1804
Anti-FLAG (SIG1-25)	Sigma	Cat# F7425
Anti-HA tag (HA.C5)	Abcam	Cat# ab18181
Anti-Myc tag (9B11)	Cell Signaling Technology	Cat# 2276
Anti-Lamin B1	Abcam	Cat# ab16048
Anti-GAPDH (1D4)	Santa Cruz Biotechnology	Cat# sc59540
Anti-rabbit IgG, HRP-linked Antibody	Cell Signaling Technology	Cat# 7074
Anti-mouse IgG, HRP-linked Antibody	Cell Signaling Technology	Cat# 7076
Goat-anti-mouse-IgG-Alexa488	Invitrogen	Cat# A11029
Goat-anti-rabbit-IgG-Alexa594	Invitrogen	Cat# A11037
**Chemicals and Recombinant Proteins**		
DAPI	Sigma Aldrich	Cat# D8417
Alexa Fluor 594 phalloidin	Invitrogen	Cat# A12381
Cyclohexamide	Sigma-Aldrich	Cat# 01810
MG132	Sigma-Aldrich	Cat# C2211
Concanamycin A	Sigma-Aldrich	Cat# C9705
Bafilomycin A1	Sigma-Aldrich	Cat# B1793
Ammonium chloride	Sigma-Aldrich	Cat# A9434
Vemurafenib (PLX4032)	Selleckchem	Cat# S1267
Ponatinib	Selleckchem	Cat# S1490
PD0325901	Sigma-Aldrich	Cat# 444966
Recombinant Human IL-6 protein	R&D systems	Cat# 206-IL-010
Recombinant Human YAP1 protein	Abcam	Cat# ab132459
Recombinant Human MAP3K3 protein	Abcam	Cat# ab132459
Lambda Protein Phosphatase	New England Biolabs	Cat# P0753S
Phosphatase inhibitor cocktail 3	Sigma-Aldrich	Cat# P0044

Phostag^{TM} acylamide	Wako Chemicals	Cat# 304-93521
Pierce^{TM} Protein G Plus Agarose	Thermofisher scientific	Cat# 22851
TRIzol^{TM} Reagent	ThermoFisher Scientific	Cat# 15596026
**Critical Commercial Assays**		
RNeasy Plus Mini Kit	Qiagen	Cat# 74134
High Capacity cDNA Reverse Transcription Kit	Applied Biosystems	Cat# 4374966
Fast SYBR Green Master Mix	ThermoFisher Scientific	Cat# 4385612
StepOne Plus real-time PCR system	Applied Biosystems	Cat# 4376357
QuickChange Lightening Site-Directed Mutagenesis Kit	Agilent	Cat# 210518
NE-PER Nuclear and Cytoplasmic Extraction Reagents	ThermoFisher Scientific	Cat# 78833
Cell Counting Kit-8	Dojindo	Cat# CK04-13
DeltaVision Spectris Imaging System	Applied Precision	N/A
FITC Annexin V Apoptosis Detection Kit	BD bioscience	Cat# 556547
BD LSRFortessa flow cytometer	BD bioscience	Cat# 649225
**Cell lines**		
RPE1, MCF-7, T47D, HEK293T and SKMEL28	ATCC	
LATS1/2-null RPE1/MCK7/HEK293T	This study	
BRAF inhibitor-resistant SKMEL28	This study	
LATS1/2-wildtype and -null HEK293A	Dr. Hyun Woo Park, Yonsei University, Korea	
**Experimental Organisms/Strains**		
Mouse: Female BALB/c nude mice	Orientbio, Gyeonggi, Korea	N/A
**RNAi Oligonucleotides**		
human MAP3K3 (#1) siRNA: GAUCUACAUUACAUGAACA	Dharmacon	N/A
human MAP3K3 (#2) siRNA: GAUAGAAGCUCAAGCAUGA	Dharmacon	N/A
human p62 (#1) siRNA: GAACAGAUGGAGUCGGAUA	Dharmacon	N/A
human p62 (#2) siRNA: GCAUUGAAGUUGAUAGCGA	Dharmacon	N/A
human p62 (#3) siRNA: GGACCCAUCUGUCUUCAAA	Dharmacon	N/A
human p62 (#4) siRNA: GGAGCACGGAGGGAAAAGA	Ambion	Cat# S16960
human MAP3K3 (#1) shRNA: CCGGGTGCGAGATCCAGTTGCTAAACTCGAGTTTAGC AACTGGATCTCGCAC TTTTTG	Sigma	Clone ID# TRCN0000002306
human MAP3K3 (#3): shRNA CCGGAGGAATACTCAGATCGGGAAACTCGAGTTTCCC GATCTGAGTATTCCT TTTTTG	Sigma	Clone ID# TRCN0000002308
**Recombinant DNA**		
FLAG-MAP3K3	Viagene	Cat# CH885110
pEGFP-C3-hYAP1	Addgene	Cat# 17843
MSCV-FLAG-YAP	Dr. Dae-Sik Lim, KAIST, Korea	N/A
pRk5-HA-Ubiquitin	Addgene	Cat# 17608
p4489 Flag-β-TrCP	Addgene	Cat# 10865

pCMV3-N-FLAG-FBXW7	Sino Biological	Cat# HG13414-NF
YAP SDM mutants	This study	N/A
MAP3K3 mutants	This study	N/A
pLKO.1-TRC cloning vector	Addgene	Cat# 10878
psPAX2	Addgene	Cat# 12260
pMD2.G	Addgene	Cat# 12259
**Software and Algorithms**		
GraphPad Prism	GraphPad	https://www.graphpad.com/
ImageJ	ImageJ	https://imageJ.nih.gov/ij/
GSEA	Broad Institute	https://software.broadinstitute.org/gsea/login.jsp
FACSDiva Software	BD Biosciences	http://www.bdbiosciences.com/kr/instruments/software/facsdiva/features/overview.jsp
R	The R Foundation	V3.2
Excel	Microsoft Office	2016

Primers

	Forward	Reverse
MAP3K3	5′ –CAGCTCAGCCCTTCTGAACA-3′	5′ –ACGCTATAATTCGCCTCTCCC-3′
CTGF	5′ –CAGCATGGACGTTCGTCTG-3′	5′ –AACCACGGTTTGGTCCTTGG-3′
CYR61	5′ –CTCGCCTTAGTCGTCACCC-3′	5′ –CGCCGAAGTTGCATTCCAG-3′
ANKRD1	5′ –AGTAGAGGAACTGGTCACTGG-3′	5′ –GGGCTAGAAGTGTCTTCAGAT-3′
KLF-2	5′ –CTACACCAAGAGTTCGCATCTG-3′	5′ –CCGTGTGCTTTCGGTAGTG-3′
YAP	5′ –CGCTCTTCAACGCCGTCA-3′	5′ –AGTACTGGCCTGTCGGGAGT-3′
IL-6ST	5′ –CACCCTGTATCACAGACTGGCA-3′	5′ –TTCAGGGCTTCCTGGTCCATCA-3′
36B4	5′ –AACATGCTCAACATCTCCCC-3′	5′ –CCGACTCCTCCGACTCTTC-3′
GAPDH	5′ –CAACGGATTTGGTCGTATTGG-3′	5′ –GCAACAATATCCACTTTACCAGAGTTAA-3′
